# Structural Changes of Inner and Outer Choroid in Central Serous Chorioretinopathy Determined by Optical Coherence Tomography

**DOI:** 10.1371/journal.pone.0157190

**Published:** 2016-06-15

**Authors:** Shozo Sonoda, Taiji Sakamoto, Nobuhiro Kuroiwa, Noboru Arimura, Hiroki Kawano, Naoya Yoshihara, Takehiro Yamashita, Eisuke Uchino, Takamasa Kinoshita, Yoshinori Mitamura

**Affiliations:** 1 Department of Ophthalmology, Kagoshima University Graduate School of Medical and Dental Sciences, Kagoshima, Japan; 2 Department of Ophthalmology, Tokushima University Graduate School of Medicine, Tokushima, Japan; Massachusetts Eye & Ear Infirmary, Harvard Medical School, UNITED STATES

## Abstract

**Purpose:**

To determine the structural changes of the choroid in eyes with central serous chorioretinopathy (CSC) by enhanced depth imaging optical coherence tomography (EDI-OCT).

**Methods:**

A retrospective comparative study was performed at two academic institutions. Forty eyes with CSC, their fellow eyes, and 40 eyes of age-matched controls were studied. Subfoveal cross sectional EDI-OCT images were recorded, and the hypo reflective and hyperreflective areas of the inner and outer choroid in the EDI-OCT images were separately measured. The images were analyzed by a binarization method to determine the sizes of the hyporeflective and hyperreflective areas.

**Results:**

In the inner choroid, the hyperreflective area was significantly larger in the CSC eyes (35,640±10,229 μm^2^) than the fellow eyes (22,908±8,522 μm^2^) and the control eyes (20,630±8,128 μm^2^; *P*<0.01 vs control for both, Wilcoxon signed-rank test). In the outer choroid, the hyporeflective area was significantly larger in the CSC eyes (446,549±121,214 μm^2^) than the control eyes (235,680±97,352 μm^2^, *P*<0.01). The average ratio of the hyporeflective area to the total choroidal area was smaller in the CSC eyes (67.0%) than the fellow eyes (76.5%) and the control eyes (76.7%) in the inner choroid (*P*<0.01, both). However, the ratio was larger in the CSC eyes (75.2%) and fellow eyes (71.7%) than in the control eyes (64.7%) in the outer choroid (*P*<0.01, both).

**Conclusions:**

The larger hyperreflective area in the inner choroid is related to the inflammation and edema of the stroma of the choroid in the acute stage of CSC. The larger hyporeflective areas in the outer choroid is due to a dilatation of the vascular lumens of the larger blood vessels. These are the essential characteristics of eyes with CSC regardless of the onset.

## Introduction

Acute central serous chorioretinopathy (CSC) is a relatively common ocular disease characterized by serous retinal detachment and/or retinal pigment epithelial (RPE) detachment. CSC occurs more often in middle aged individuals and tends to regress spontaneously in several months although it frequently recurs. Evidence indicates that vascular abnormalities of the choroid play important roles in the pathogenesis of CSC [1]. This is confirmed by the delayed filling and hyperpermeability of the choroidal vessels detected in the fluorescein angiograms of eyes with CSC [1–3]. The hyperpermeability of the choroidal vessels is believed to increase the tissue hydrostatic pressure which can then cause retinal pigment epithelial (RPE) detachment. This would then lead to a breakdown of the barrier function of the RPE resulting in fluid leakage into the submacular space. However, the exact pathogenesis of CSC has yet to be determined [1].

Optical coherence tomographic examinations of eyes with acute CSC have shown that the choroid is significantly thickened, and the thickness gradually decreased as the CSC regressed [3–10]. The choroid was thicker in areas with punctate hyperfluorescent spots than in unaffected areas in the indocyanine angiograms (IA). However, there are other reports that state that the areas of IA staining did not correspond with areas of choroidal thickening in the optical coherence tomographic (OCT) images [11,12]. Thus, whether the choroid is thickened in eyes with CSC remains to be conclusively determined.

While it is relatively easy to determine whether the choroid is thickened, it is difficult to determine which structures or areas of the choroid are thickened. Unfortunately, the choroid is not well-organized morphologically which makes it difficult to detect which structures are altered in eyes with CSC. Yet this information is essential for determining the mechanism causing the choroidal thickening.

We have developed a binarization method for differentiating the hyperreflective and hyporeflective areas of the choroid in the OCT images quantitatively [13,14]. Although it has not been conclusively proven, a comparison to the histological and empirical results strongly suggest that the hyperreflective areas represent the stromal areas and the hyporeflective areas the vascular luminal or fluid-filled areas in normal eyes. Thus, this binarization method allows quantitative analyses of the stroma and vascular areas objectively with high repeatability and reproducibility non-invasively [13,14].

The purpose of this study was to determine the changes in the stromal and luminal areas of the choroid in eyes with CSC. To accomplish this, we used the binarization method and determined the hypo reflective and hyperreflective areas of the inner and the outer choroid in the subfoveal area of eyes with CSC. We shall show that there are unique structural changes of the two areas of the choroid in the acute stage of CSC.

## Methods

This was a retrospective clinical study of one eye of each subject. All of the procedures conformed to the tenets of the Declaration of Helsinki. A written informed consent was obtained from all of the subjects. The study was approved by the Ethics Committees of the Kagoshima University Hospital (Kagoshima, Japan) and the Tokushima University Hospital (Tokushima, Japan). This study was registered with the University Hospital Medical Network (UMIN)-clinical trials registry (CTR). The registration title is “UMIN000012310, Choroidal structure on OCT images”. A detailed protocol is available at https://upload.umin.ac.jp/cgi-open-bin/ctr/ctr.cgi?function=brows&action=brows&type=summary&recptno=R000014386&language=J.

All subjects were diagnosed with acute CSC at the Kagoshima University Hospital, Kagoshima, Japan or the Tokushima University Hospital, Tokushima, Japan, between December 1, 2013 and October, 2014. The diagnosis of acute CSC was made by the presence of; neurosensory retinal detachment at the macula, one or more leaking spots from the RPE at the acute stage, and a late expansion of the leaks with typical smoke stack-shaped fluorescein angiograms (FA). Eyes with other diseases such as epiretinal membrane and age-related macular degeneration, or eyes with previous treatments were excluded. Myopic eyes of -6 diopters (D) or higher were also excluded. When both eyes were affected, the cases were excluded because the normal fellow eyes were used as controls. Individuals without any eye disease and age-matched to the CSC group (± 1 year) were used as controls and only the right eye was studied.

Prior to the OCT recordings, all of the eyes had a comprehensive ocular examination including slit-lamp examinations of the anterior segment of the eye and ophthalmoscopic examinations of the fundus. The intraocular pressure was measured with a pneumotonometer (CT-80, Topcon, Tokyo, Japan), and the axial length was measured with the AL-2000 ultrasound instrument (Tomey, Tokyo, Japan). The best-corrected visual acuity (BCVA) was measured after determining the refractive error with an Auto Kerato-Refractometer (RM8900, Topcon).

### Fluorescein angiography (FA) and indocyanine green angiography (IA)

Standard FA and IA were performed on all patients without allergy to fluorescein and indocyanine green with the Heidelberg angiographic instrument (HRA2, Heidelberg Engineering, Heidelberg, Germany). The angiograms were evaluated by two independent observers (SS, HK) who were masked to the clinical findings. To determine whether pathological findings were present in the choroid, special attention was paid to the presence of hyperpermeability in the IAs. If the interpretation was split between the two graders, a third grader (NA) decided the diagnosis.

### Choroidal images measured by spectral-domain optical coherence tomography (SD-OCT)

The choroidal tomographic images were obtained by enhanced depth imaging (EDI)-OCT as described in detail in our earlier reports [13,14]. The scans were 7 horizontal lines of 30 x 10 degrees through the center of the fovea made with a Heidelberg Spectralis OCT (Spectralis; Heidelberg Engineering) instrument. Each image was obtained using the eye tracking system, and 100 scans were averaged. All eyes were examined without mydriasis, and each eye was measured twice within 1 hour. The OCT images were analyzed by two independent examiners (NK, NY). Because there are significant diurnal fluctuations of the choroidal thickness, all examinations on a single patient were done within one hour on the same day from 11:00 to 13:00 hours.

### Evaluation of total choroidal, and hyporeflective and hyperreflective areas by binarization technique

Recording the OCT images and their processing were performed as reported in detail [13,14]. Briefly, the best EDI-OCT image was displayed on a computer screen and evaluated by 3 masked graders independently. When 2 or more graders determined that the choroidal image was clear, the image was deemed acceptable and used for the following analyses.

The upper margin of the region of interest (ROI) was the RPE line and the lower margin was the chorioscleral border. The OCT images were saved in the TIFF format and analyzed with the ImageJ software (ImageJ version 1.47, National Institutes of Health, Bethesda, MD; available at: http://imagej.nih.gov/ij/). The subfoveal area of the choroid analyzed extended from the RPE to the chorioscleral border and from 750 μm nasal and 750 μm temporal to the fovea. Then three choroidal vessels with lumens larger than 100 μm were randomly selected by the Oval Selection Tool on the ImageJ tool bar, and the average reflectivity of these areas was determined in RGB images. The average brightness was set as the minimum value to minimize the noise in the OCT image.

**Fig 1:**
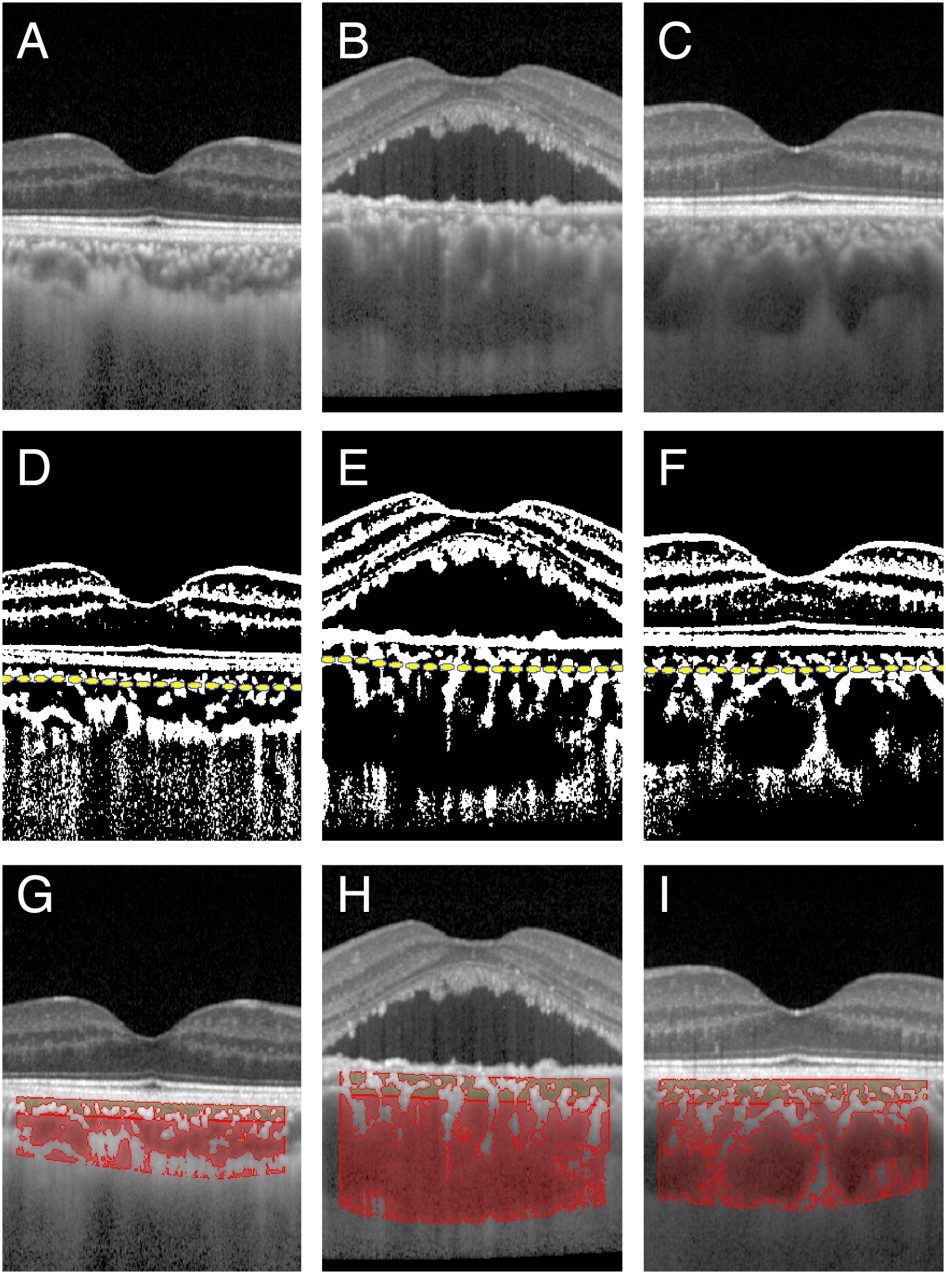
Spectral domain optical coherence tomographic (SD-OCT) images of an eye with central serous chorioretinopathy, of the normal fellow eyes, and normal control eyes. SD-OCT images of a control eye (A, D and G), an eye with central serous chorioretinopathy (CSC; B, E, and H), and the fellow eyes of a patient with unilateral CSC (C, F, and I) are shown. The original OCT images (A-C), binarized images (D-F), and the images in which the hyporeflective areas are outlined in color are shown (G-I). Yellow dashed lines indicate the segmentation lines between the inner and outer choroid (middle row). The area outlined in dark yellow represents the hyporeflective area in the inner choroid and the one in dark red represents the hyporeflective area in the outer choroid.

Then, the original RGB image was converted to 8 bits and adjusted by the Niblack auto local threshold method (Fig 1). If the Niblack method does not appear, a plug-in link is needed. This link can be downloaded at: http://www.mecourse.com/landinig/software/auto_threshold.jar. After considering the distance between two pixels, the areas of the total choroid, the hyporeflective, and hyperreflective areas of the outer and inner choroid were automatically determined.

The margins of the inner and the outer choroid was determined by the Branchini et al method with some modifications [15,16]. Because our preliminary study showed that the inter-rater agreement by the original Branchini’s method was not high, the segmentation was done on the binarized OCT images as follows. Lines were drawn perpendicular to the RPE layer from the inner most point of 5 randomly selected large (≥100 μm) luminal areas within the ROI. The average length of these 5 lines was defined as the border between the inner choroidal vascular layer and the outer choroidal layer. The inner choroidal layer included the choriocapillaris and Sattler’s layer (Fig 1). The intra-rater agreement (NK) and inter-rater agreement (NK and YN) of the measurements of the entire choroidal area, hyporeflective area, and hyperreflective area of each area were determined.

### Central serous chorioretinopathy (CSC) index

Our preliminary study showed that the structural changes of the inner and outer choroid in eyes with CSC differed so we developed a new index, the CSC index, to quantify the differences. The formula to determine the CSC index was:

$$CSC\;index=\frac{\;\frac{hyporeflective\;area\;of\;outer\;choroid}{hyperreflective\;area\;of\;outer\;choroid}\;}{\frac{hyporeflective\;area\;of\;inner\;choroid}{hyperreflective\;area\;of\;inner\;choroid}}$$

### Choroidal structure and IA finding

The correlations between the degree of hyperpermeability and the different choroidal structures were calculated [1–3]. The video of the IA was projected onto a screen, and the presence of hyperpermeability was determined within a 1500 μm area which included the fovea, by two independent examiners (SS, HK) who were masked to the results of OCT. The presence of hyperpermeability in the IA was determined as described in earlier reports [1–3]. A third examiner was consulted when the diagnosis was split. Each eye was graded into 4 degrees of hyperpermeability; very severe, severe, moderate, and mild based on the IA findings. Then, the eyes classified as having high hyperpermeability were placed in one group and the other eyes in the low hyperpermeability group. The correlation of each choroidal area and the two permeability groups was determined.

### Statistical analyses

All statistical analyses were performed with SPSS statistics 19 for Windows (SPSS Inc., IBM, Somers, NY). The significance of the differences in the total cross sectional choroidal area, the luminal area, the stromal area, the luminal/stromal ratio, and the CSC index in the two groups were determined by the Wilcoxon signed-rank test with Bonferroni correction. The significance of differences of the baseline characteristics of each parameter in each group was determined by χ^2^ test; e.g., the sex distribution of the subjects, or the Mann-Whitney U test for the age, refractive error, visual acuity, and intraocular pressure. The correlation of each choroidal area and the difference in the extent of choroidal hyper-permeability was determined by the Mann-Whitney U tests. The intra-rater correlation coefficients were calculated by 1-way random effects model for measurements of agreement. The inter-rater correlation coefficient was calculated using a 2-way mixed-effects model for measurements of absolute agreement. A *P* of <0.05 was considered to be statistically significant.

## Results

### Demographic data

Forty-seven eyes with CSC were studied. Among them, 4 eyes were excluded because the CSC was bilateral, 2 eyes because of a history of CSC in the fellow eyes, and 1 eye with age-related macular degeneration. In the end, 40 eyes with CSC and their normal fellow eyes, and 40 age-matched normal control eyes were studied. There were no significant differences in the sex distribution, age, and IOP between the CSC eyes and the control eyes (Table 1). It is also described in S1 and S2 Tables).

**Table 1:** Demographics of studied eyes.

	Sex (M/F)	Age (years)	Refractive error (diopter)**	LogMAR BCVA**	IOP (mmHg)
Control N = 40	26/14	45.63 ± 8.09	-2.13 ± 1.82	-0.14 ± 0.08	13.20 ± 2.21
CSC N = 40	31/9	45.55 ± 8.16	-0.16 ± 1.34	0.04 ± 0.18	12.97 ± 2.43
CSC fellow eye N = 40	31/9	45.55 ± 8.16	-1.09 ± 1.50	-0.08 ± 0.07	12.83 ± 2.79
P-value^a^	P = 0.22	P = 0.92	Note 1	Note 1	Note 2

CSC, central serous chorioretinopathy; logMAR, logarithm of the minimum angle of resolution; BCVA, best corrected visual acuity; IOP, intraocular pressure.

^a^ χ^2^ test or Mann-Whitney-U test.

** and Note 1: Significant difference between any two of three groups (P<0.01).

Note 2: No significant difference between any two of three groups.

The visual acuity was significantly better in the control eyes than in the CSC eyes, (*P* <0.01. Mann-Whitney U test). The mean BCVA of the fellow eyes was -0.08 ± 0.07 logMAR units which was significantly better than that of the CSC eyes at 0.04 ± 0.18 logMAR units (*P* <0.01, Mann-Whitney U test). The mean refractive error of the fellow eyes was -1.09 diopters which was significantly more myopic than that of the CSC eyes at -0.16 ± 1.3 diopters (*P* <0.01, Mann-Whitney U test).

### Choroidal areas

**Fig 2:**
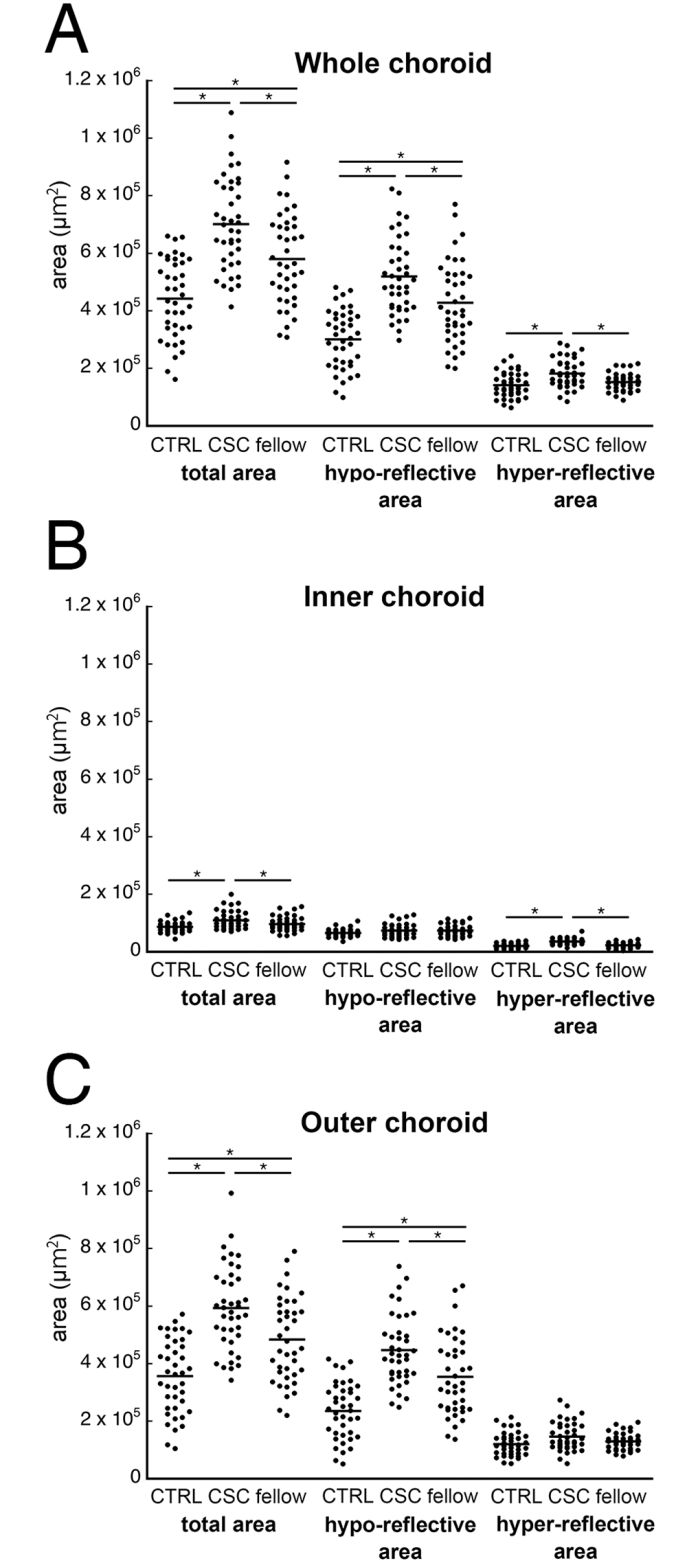
Scatterplots of choroidal areas of control eyes, CSC eyes, and fellow eyes. The total choroidal area, hyporeflective area, and hyperreflective area in the three types of eyes are shown as a function of the location in the choroid. (A) whole choroid, (B) inner choroid, and (C) outer choroid. *; *P* <0.01, Bonferroni’s correction by Wilcoxon signed-rank test.

A scatter plot of the areas of the choroid, hyporeflective regions, and hyperreflective regions as a function of the location are shown for each eye in Fig 2. The intra-rater agreement of the value of the choroidal area was high for the CSC eyes (Table 2).

**Table 2:** Intra-rater agreement of measurement of choroidal area in central serous chorioretinopathy.

	CSC		P value*
	ICC	95% CI	
Whole choroid			
total area	0.997	(0.995–0.999)	P<0.01
hypo-reflective area	0.997	(0.995–0.999)	P<0.01
hyper-reflective area	0.974	(0.952–0.986)	P<0.01
Inner choroid			
total area	0.988	(0.977–0.994)	P<0.01
hypo-reflective area	0.979	(0.961–0.989)	P<0.01
hyper-reflective area	0.917	(0.850–0.955)	P<0.01
Outer choroid			
total area	0.996	(0.993–0.998)	P<0.01
hypo-reflective area	0.996	(0.993–0.996)	P<0.01
hyper-reflective area	0.968	(0.941–0.983)	P<0.01

CSC, central serous chorioretinopathy; CI, confidence interval; ICC, intraclass correlation coefficients.

* *P* < 0.05.

Although the inter-rater agreement was high for the CSC eyes, fellow eyes, and control eyes, the intra-class correlation coefficients were slightly lower for the inner choroidal than the outer choroidal areas (Table 3). The average and standard deviations (SDs) of each area is shown in Table 4. (S3, S4 and S5 Tables)

**Table 3:** Interrater agreement of measurement of choroidal area.

	Controls		CSC		CSC-fellow		P-value
	ICC	95% CI	ICC	95% CI	ICC	95% CI	
Whole choroid							
total area	0.991	(0.983–0.991)	0.984	(0.970–0.985)	0.969	(0.928–0.970)	P<0.01
hypo-reflective area	0.992	(0.985–0.992)	0.985	(0.971–0.985)	0.977	(0.959–0.979)	P<0.01
hyper-reflective area	0.976	(0.956–0.977)	0.952	(0.912–0.954)	0.974	(0.950–0.975)	P<0.01
Inner choroid							
total area	0.886	(0.790–0.983)	0.92	(0.840–0.920)	0.916	(0.832–0.922)	P<0.01
hypo-reflective area	0.924	(0.849–0.919)	0.962	(0.930–0.964)	0.954	(0.911–0.953)	P<0.01
hyper-reflective area	0.876	(0.781–0.877)	0.908	(0.815–0.910)	0.912	(0.822–0.922)	P<0.01
Outer choroid							
total area	0.99	(0.981–0.994)	0.98	(0.963–0.981)	0.986	(0.988–0.997)	P<0.01
hypo-reflective area	0.992	(0.984–0.992)	0.981	(0.965–0.990)	0.988	(0.922–0.994)	P<0.01
hyper-reflective area	0.971	(0.946–0.972)	0.956	(0.919–0.958)	0.984	(0.970–0.985)	P<0.01

**Table 4:** Choroidal area on EDI-OCT images.

	total area					hypo-reflective area					hyper-reflective area				
	mean (SD) μm^2^		Sig. difference			(SD) μm^2^		Sig. difference			mean (SD) μm^2^		Sig. difference		
Ctrl	442877	(137153)	*	*		301696	(101640)	*	*		141181	(43339)	*		
CSC	702101	(160652)	*		*	520020	(132420)	*		*	182081	(49484)	*		*
Flw	580833	(155547)		*	*	428276	(141512)		*	*	152557	(29996)			*
Ctrl	86646	(18200)	*			66016	(12998)				20630	(8128)	*		
CSC	109111	(30665)	*		*	73471	(22797)				35640	(10229)	*		*
Flw	96730	(24579)			*	73822	(19008)				22908	(8522)			*
Ctrl	356231	(128928)	*	*		235680	(97352)	*	*		120551	(40045)			
CSC	592990	(146632)	*		*	446549	(121214)	*		*	146441	(49822)			
Flw	484103	(146470)		*	*	354455	(135816)		*	*	129648	(28847)			

SD, standard deviation; Sig. difference, Statically significant difference was found between two groups in the same column.

* P<0.01, Bonferroni’s correction by Wilcoxon signed-rank test.

CSC, central serous chorioretinopathy; Ctrl, control eyes; Flw, fellow eyes of CSC eyes; n = 40 in each group.

#### CSC eyes and control eyes

The mean total cross sectional area of the subfoveal choroid of the CSC eyes was significantly larger than that of the control eyes (*P* <0.01, Wilcoxon signed-rank test). The mean hyporeflective area of the total choroid was significantly larger in the CSC eyes than in the control eyes (*P*<0.01). The mean hyperreflective area of the total choroid was also significantly larger in the CSC eyes than in the control eyes (*P* <0.01). The mean inner choroidal area was significantly larger in the CSC eyes than in the control eyes (*P* <0.01). The mean hyperreflective area of the inner choroid was significantly larger in the CSC eyes than in the control eyes (*P* <0.01), but the mean hyporeflective area in the inner choroid of the CSC eyes was not significantly larger than that of the control eyes (*P* = 0.21). The mean outer choroidal area was significantly larger in the CSC eyes than in the controls eyes (*P* <0.01). The mean hyporeflective area of the outer choroid was significantly larger in the CSC eyes than in the control eyes (*P* <0.01). In contrast, the mean hyperreflective area in the outer choroid of the CSC eyes was not significantly larger than in the control eyes (*P* = 0.02; Table 4).

**Fig 3:**
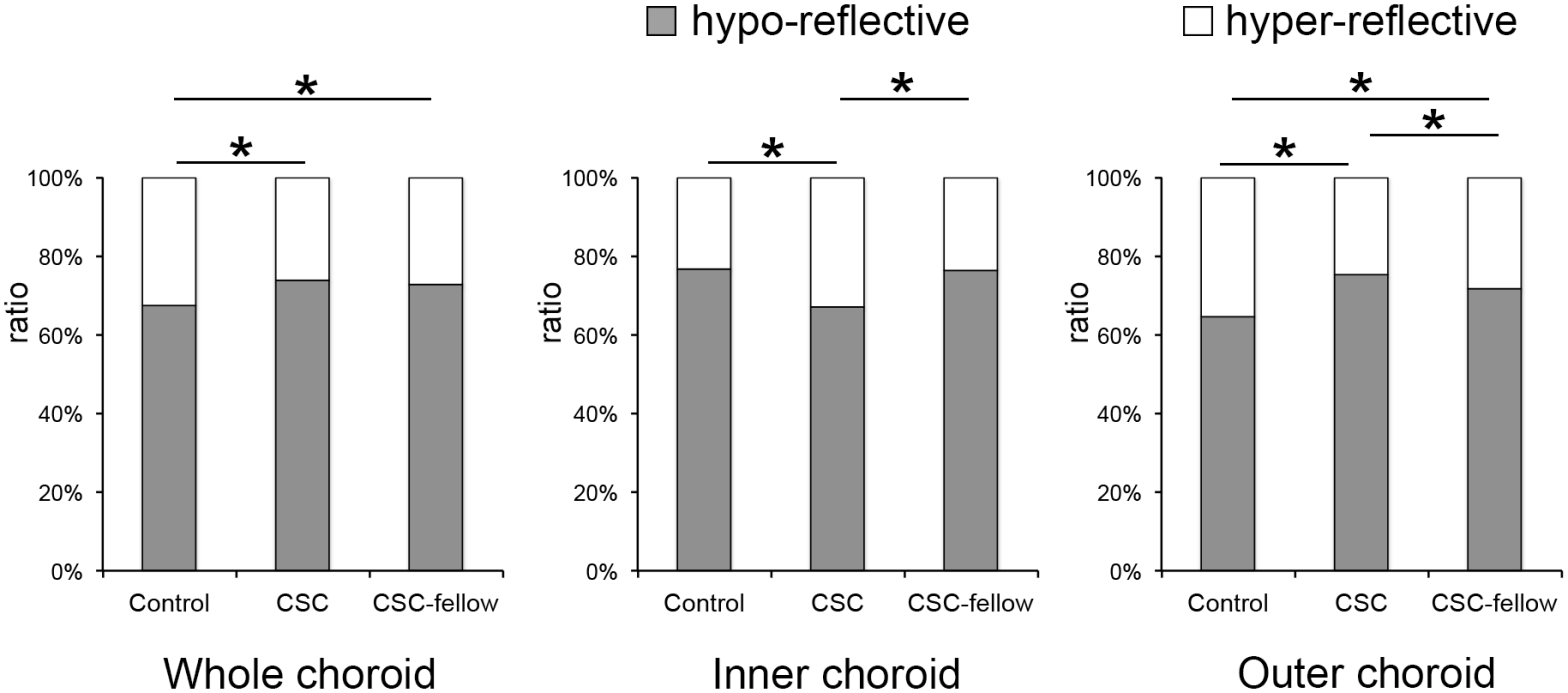
The ratio of hyporeflective and hyperreflective areas in the choroid. The total choroidal area (left), the inner choroidal area (center), and the outer choroidal area (right) are shown. The ratio of hyporeflective areas was significantly larger in CSC eyes in the total choroid or CSC fellow eyes. But it was significantly smaller in CSC eyes than fellow eyes or control eyes. *; *P*<0.01, Wilcoxon signed-rank test; CTRL, control eyes.

The ratios of each area of the choroid are shown in Fig 3. For the total subfoveal choroidal area, the ratio of the hyporeflective areas was significantly larger in the CSC eyes than in the control eyes (*P* <0.01), and the same was also true for the outer choroidal area. However, the ratio of hyporeflective area to the corresponding choroidal area in the inner choroid was significantly smaller in the CSC eyes than in the control eyes (*P* <0.01).

#### CSC eyes and fellow eyes

The mean of the cross sectional area of the total subfoveal choroid in CSC eyes was significantly larger than that of the fellow eyes (*P* <0.01, Wilcoxon signed-rank test). The means of the hyporeflective and hyperreflective areas of the total choroid in the CSC eyes was significantly larger than that of the fellow eyes (both *P* <0.01). The mean inner choroidal area was significantly larger in the CSC eyes than in the fellow eyes (*P* <0.01), but the mean hyporeflective area in the CSC eyes was not significantly larger than that of the fellow eyes (*P* = 0.93). The mean hyperreflective area was significantly larger in the CSC eyes than in the fellow eyes (*P* <0.01). The mean outer choroidal area was significantly larger in the CSC eyes than in the fellow eyes (*P* <0.01), and the mean hyporeflective area in the outer choroid was significantly larger in the CSC eyes than in the fellow eyes (*P* <0.01). However, the mean hyperreflective area of the outer choroid was not significantly larger in CSC eyes than the fellow eyes (*P* = 0.08; Table 4).

The ratio of each component is shown in Fig 3. For the total subfoveal choroidal area, the ratio of hyporeflective area of the CSC eyes to the corresponding choroidal area of the fellow eyes was not significantly larger in the CSC eyes than in the fellow eyes (*P* = 0.30). The ratio of hyporeflective area to the corresponding choroidal area in the inner choroid was significantly smaller in the CSC eyes than in the fellow eyes (*P* <0.01). On the other hand, the ratio of the hyporeflective area to the corresponding choroidal area in the outer choroid was significantly larger in the CSC eyes than in the fellow eyes (*P* = 0.016).

#### Fellow eyes and control eyes

The mean of the total choroidal area of the fellow eyes was significantly larger than that of the control eyes (*P* <0.01, Wilcoxon signed-rank test). The mean of the hyporeflective area of the total choroid was also significantly larger in the fellow eyes than in the control eyes (*P* <0.01). However, the mean hyperreflective area of the total choroidal area was not significantly larger in the fellow eyes than in the control eyes (*P* = 0.09). The inner choroidal area was not significantly different between the fellow eyes and control eyes (*P* = 0.04, Bonferroni’s correction). The mean hyporeflective area and the hyperreflective area of the inner choroidal area of the fellow eyes were not significantly different from that of the control eyes (*P* = 0.06 and *P* = 0.25, respectively). The mean hyporeflective area of the outer choroidal area of the fellow eyes was significantly larger than that of the control eyes (*P* <0.01), however, the hyperreflective area was not significantly different between the two groups (*P* = 0.10) (Table 4).

The ratios of each component are shown in Fig 3. For the total subfoveal choroidal area, the ratio of the hyporeflective area to the corresponding choroidal area was significantly larger in the fellow eyes than in the control eyes (*P* <0.01). For the inner choroid, the ratio of the hyporeflective area to the corresponding choroidal area was not significantly larger in fellow eyes than in the control eyes (*P* = 0.60). For the outer choroid, the ratio of the hyporeflective area was larger in the fellow eyes than the control eyes (*P* <0.01).

### CSC index

**Fig 4:**
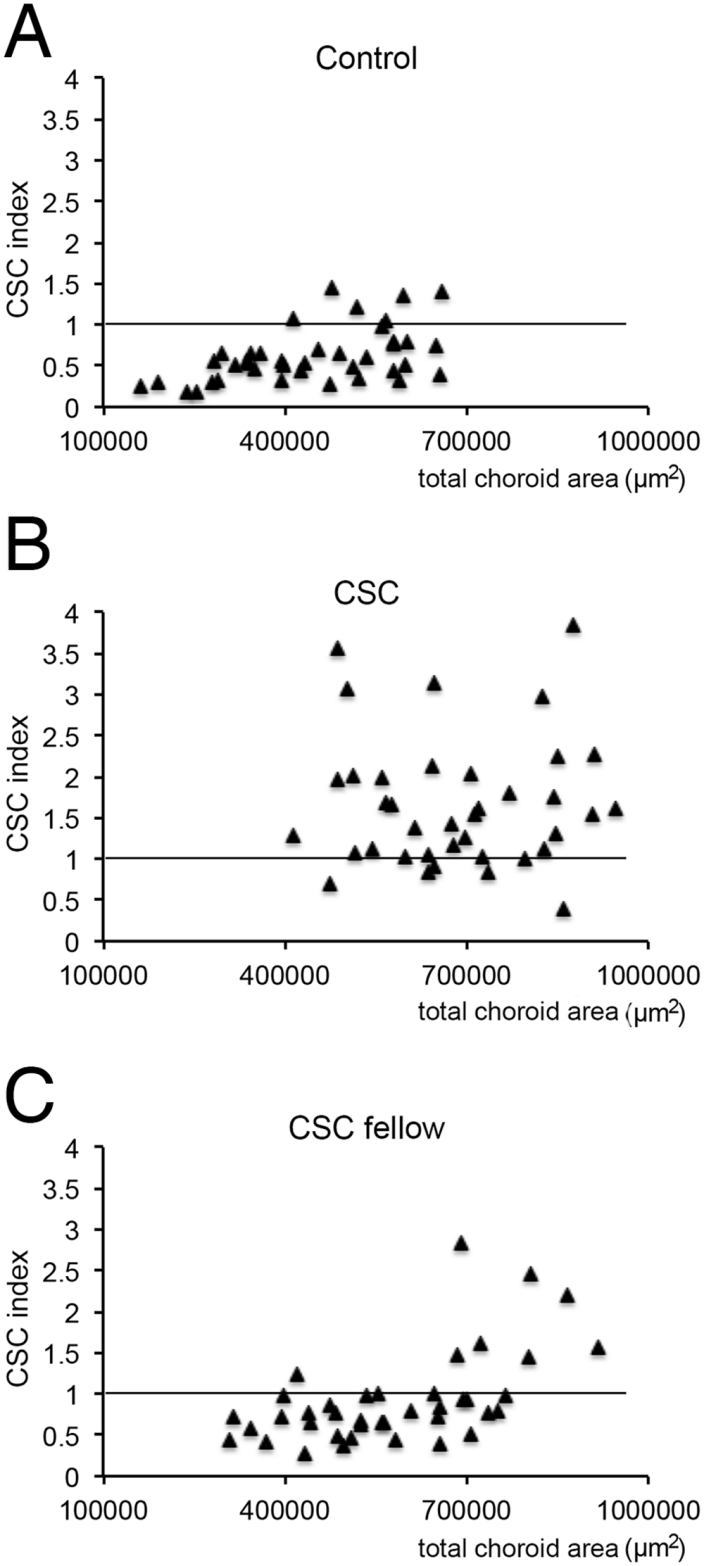
Scatterplots of CSC index as a function of the total choroidal area for each type of eyes. Scatterplot of the eyes for the total choroidal area (X-axis) and CSC index (Y-axis) is shown. In controls, the choroidal area was distributed at a lower range than CSC eyes, and the CSC index was distributed at a lower range than CSC eyes (A, B). CSC-fellow eyes were between these two groups from either point (C).

A scatterplot of the choroidal thickness and CSC index is presented in Fig 4. A CSC index of 1.0 indicates that the ratio of the hyporeflective area of the inner choroid is equal to that of the outer choroid, and if the index is greater than 1.0, the ratio of the hyporeflective area of the outer choroid is larger than that for the inner choroid.

For the control eyes, the total choroidal area ranged from 161,950 to 659,849 μm^2^, and the mean CSC index was 0.62. Six of 40 eyes (15.0%) of the controls had CSC index greater than 1.0. But in the CSC eyes, the choroidal area was larger than 413,998 μm^2^. The mean CSC index was 1.70, and 35 of 40 eyes (87.5%) of the CSC eyes had a CSC index greater than 1.0. In the fellow eyes, the choroidal area was distributed mostly in the zone higher than that of the controls. However, distribution of CSC index was similar to that of controls rather than CSC eyes; a mean CSC Index was 0.93 and 8 eyes of 40 (20.0%) of fellow eyes had a CSC index greater than 1.0. No significant difference was found between higher and lower CSC index groups in the CSC fellow eyes.

### Choroidal structure and IA findings

Clear IA images were obtained from 29 eyes with CSC, and 14 eyes were classified as having high hyperpermeability and 15 eyes with low hyperpermeability. There were no significant differences in the total choroidal area, hyporeflective area, or the hyperreflective area between the 2 groups (Table 5).

**Table 5:** Indocyanine green angiographic findings and choroidal area.

	IA-HP high (n = 14)		IA-HP low (n = 15)		P value
	average (SD) μm^2^		average (SD) μm^2^		
Whole choroid					
total area	717368	(157608)	667026	(183437)	0.19
hypo-reflective area	534562	(135374)	479119	(130320)	0.22
hyper-reflective area	182805	(48101)	187906	(61064)	0.9
Inner choroid					
total area	106814	(25145)	100641	(19803)	0.51
hypo-reflective area	69421	(20619)	66890	(15717)	0.97
hyper-reflective area	37393	(6494)	33750	(7549)	0.18
Outer choroid					
total area	610553	(148324)	566385	(175719)	0.21
hypo-reflective area	465141	(129178)	412229	(124171)	0.34
hyper-reflective area	145412	(47689)	154156	(58810)	0.69

## Discussion

We measured the hyporeflective area and hyperreflective area of the choroid in the OCT images separately. Many empirical findings support the idea that the hyperreflective areas represent non-fluid tissues such as the stroma. However recently, Spaide and Ryan reported that there was a hyporeflective location of fluid in the outer choroid of CSC eyes [17]. Therefore, it is not necessarily exact to call the hyporeflective areas the “luminal areas” in CSC eyes.

Nevertheless, the total subfoveal choroidal area in the CSC eyes was significantly larger than that of the fellow and control eyes which is consistent with earlier reports [6,7,18]. On the other hand, the ratio of outer choroid of the CSC eyes to that of the control eyes was 166% which was significantly larger than that of the inner choroid (126% of normal eyes). In the outer choroid, the hyporeflective area was significantly larger in CSC eyes than in the control eyes (189% of control eyes). In contrast, the hyperreflective area stromal area of the outer choroid was just 121% of that of controls. Therefore, the thickening of the choroid in the CSC eyes is mainly due to the larger hyporeflective area of the outer choroid. This is probably due to the dilatation of the blood vessels which agrees with the earlier reports that the lumen of the choroidal vessel appeared dilated in CSC eyes [4,19,20]. Another possibility is that the presence of fluid in a loculus [17]. Unfortunately, the present method cannot distinguish between the lumens of vessels and a loculus of fluid but these two changes might exist simultaneously in CSC eyes.

The total area of the inner choroid of the CSC eyes was significantly larger than that of the fellow eyes or the control eyes. The hyporeflective area of the CSC eyes was not significantly different from that of the CSC fellow eyes and control eyes. However, the hyperreflective area of the CSC eyes was significantly larger than that of the fellow eyes or control eyes. The hyperreflective areas probably represent the non-fluid areas such as the choroidal stroma. The choroidal stroma is made up of the vascular walls, melanocytes, fibrocytes, and neural tissues. The area of the stroma can be changed by edema and infiltration by inflammatory cells. Considering the acute nature of CSC, the stromal swelling is probably due to increased permeability of the vessels or inflammation. In general, the increased permeability leads to elevated hydrostatic pressure causing edema of the stroma. This is supported by the hyperfluorescence or leakage of indocyanine green dye in CSC eyes. However, the analysis of the results did not find a significant correlation between the IA findings and choroidal structure in the OCT images.

It is also important to note that the ratio of the hyporeflective area/total choroidal area of the CSC eyes (73.9%) and the fellow eyes (72.7%) was larger than that of the control eyes (67.5%, Fig 3). For the outer choroid, the ratio for the CSC eyes was 75.2% and that for the fellow eyes was 71.7% which are significantly larger than that of normal eyes (64.7%). These findings indicate that the choroidal structures of both eyes of CSC patients are most likely different from that of normal eyes. In the inner choroid, although the hyporeflective area was not different among the groups, the hyperreflective area, the stromal area, was significantly larger in the CSC eyes than the fellow eyes resulting in a lower ratio of the hyporeflective area (67.0% for CSC eyes, 76.5% for the fellow eyes, and 76.7% for the control eyes; Table 4). These findings are probably signs that the acute CSC was progressing. This trend was also noted in the scatterplots of the CSC index and choroidal area **(**Fig 4**)**. The distribution of the choroidal areas of the CSC eyes was similar to that of the fellow eyes. However, the distribution of the CSC index of the CSC eyes was different from that of the fellow eyes or control eyes. These structural differences may be associated with the activation of the disease process. If so, the shift of the CSC index in a single eye may be a predictor of a recurrence of acute CSC.

The concept of structural uniqueness of the choroid in CSC patients is supported by the biometrically unique characteristics of CSC eyes and frequent recurrences [21]. When CSC develops, the increase in the volume of the stroma of the inner choroid would lead to an increase of hydrostatic pressure and fluid leakage into the subretinal space through the damaged RPE cells. CSC is often the result of impaired choroidal vascular autoregulation by steroids, catecholamines, or sympathomimetic agents [1]. A monkey CSC model produced by repeated intravenous injections of epinephrine showed damage to the choriocapillaris endothelium [5]. In that model, extensive fibrin leakage into the stroma from the choriocapillaris was reported. Similar events may occur in the eyes of patients with CSC. These findings can be a cause or a result.

There are several reports on the choroidal blood flow in CSC eyes [20,22,23]. Laser Doppler flowmetry has shown that the blood flow of the inner choroidal vessels was decreased in CSC eyes [23]. Even though the influx volume is static, the blood flow can be reduced within the hyperpermeable vessels in the edematous tissues with elevated hydrostatic pressure [2,22]. In contrast, the blood flow of the deep choroidal vessels was increased in eyes with active CSC [24]. The investigators suggested that the impeded blood overflowed into the larger choroidal vessel layer through arteriovenous shunts leading to the increased blood flow [24]. There are opposite interpretations from IA studies that state that vascular dilatation is due to blood congestion [4,20]. These contradictory findings require further investigations.

The strength of the present study is that the structure of the choroid was measured quantitatively. The measurements of the hyporeflective and hyperreflective areas were done automatically so that the results are essentially objective. At the beginning of this study, we used the original Branchini’s method to segment the choroid [15], but this method was best to segment a single point of depth of the large vascular layer of the choroid. However when measuring an area, the interclass coefficient was not high at less than 0.7 in our preliminary studies. In fact, it was difficult to determine the border of the choroidal vessels on the gray scale OCT images with good repeatability. Even a minimal segmentation difference, e.g., several pixels, at a single point, made a large difference in the results because the data of various points are required in measuring the area. Thus, we used the binarized images. As a result, the intraclass coefficients improved to ≥0.917 which was sufficiently high. This method required only a commercially available OCT instrument with publically available ImageJ. Therefore, the validation of these results on CSC can be done by most researchers.

There are clear limitations in this study. A retrospective study cannot avoid sampling bias. Second, manual segmentation cannot achieve perfect reproducibility, and a truly objective method would be preferable. Third, the signal-to-noise ratio was not good enough in the outer choroid so that the interpretation of outer choroid should be done carefully. Our preliminary study showed that the hyporeflective area in the present binarized image did not significantly differ from the results with swept source-OCT in which the OCT signal is not attenuated even in the choroid. However, those limitations should be remembered when interpreting the results especially of the outer choroid. Most importantly, the finding of inner choroid was not affected by the choroidal thickness.

In conclusion, the results show that there are structural changes of the choroid in eyes with CSC, e.g., the larger hyperreflective area in the inner choroid and the larger hyporeflective area in the outer choroid. Most importantly, the quantification of the structural components of the choroid in the OCT images has extracted new information which should contribute to the determination of the mechanism of CSC.

## Supporting Information

S1 Table Demographic findings of CSC eye and CSC fellow eye. (PDF)

S2 Table Demographic findings of control eye. (PDF)

S3 Table Choroidal area of CSC eye. (PDF)

S4 Table Choroidal area of CSC fellow eye. (PDF)

S5 Table Choroidal area of control eye. (PDF)
