# Supplementary material for: Structural Changes of Inner and Outer Choroid in Central Serous Chorioretinopathy Determined by Optical Coherence Tomography
Source: PLoS One. 2016 Jun 15;11(6):e0157190. doi: 10.1371/journal.pone.0157190 (PMC4909210; doi:10.1371/journal.pone.0157190)
Supplement: S1 Table — (PDF) [file pone.0157190.s001.pdf]

Supplementary Data

S1 Table. Demographic findings of CSC eye and CSC fellow eye.

			CSC eye				CSC fellow eye		
Case number	sex	age	Refractive error	VA logMAR	IOP		Refractive error	VA logMAR	IOP
CSC-1	m	36	0.5	0	13		-0.5	0	12
CSC-2	f	37	0	-0.18	16		-2	-0.17609	15
CSC-3	m	38	-0.5	0.3	10		-2.5	0.04576	11
CSC-4	m	39	-0.5	0.22	18		-3	-0.07918	18
CSC-5	m	39	1.5	0.05	12		-0.5	-0.17609	11
CSC-6	m	40	0.75	-0.08	13		-1.25	-0.17609	3
CSC-7	f	40	-5	-0.08	17		-6	0	18
CSC-8	m	41	-0.75	-0.08	9		-2	-0.07918	10
CSC-9	m	41	1.25	0.05	13		-0.5	-0.17609	13
CSC-10	m	42	0.25	-0.08	14		-1	0	14
CSC-11	f	43	-3.75	-0.08	13		-5.25	-0.07918	13
CSC-12	m	43	0	-0.08	10		-0.5	-0.17609	10
CSC-13	m	43	0	-0.18	12		-1	-0.07918	11
CSC-14	f	44	0	0.7	13		-0.25	0	16
CSC-15	m	45	1.5	0	8		-0.25	0	10
CSC-16	m	47	-0.5	0	16		-0.5	-0.17609	16
CSC-17	f	48	-0.5	0.05	17		-1.25	-0.07918	16
CSC-18	f	50	0.5	0	13		-0.5	-0.17609	14
CSC-19	m	51	0.5	-0.18	14		-1	-0.07918	12
CSC-20	m	54	-1.75	0.15	11		-1.75	0	14
CSC-21	m	60	0	0.4	10		-1.5	-0.07918	11

CSC-22	m	64	1.25	0.22	15		-0.25	-0.17609	11
CSC-23	m	44	0.5	0.3	15		0	-0.07918	14
CSC-24	m	49	-1	0.1	11		-1.5	0	11
CSC-25	m	50	0.5	0.05	10		-0.25	-0.07918	10
CSC-26	f	52	-1.5	0	13		-1	-0.07918	12
CSC-27	m	50	-0.25	0.22	14		-1.25	0	12
CSC-28	m	52	3	0.05	11		3.25	-0.17609	12
CSC-29	m	42	0.25	-0.08	13		0.25	-0.07918	15
CSC-30	f	32	0	-0.08	14		-0.25	-0.07918	14
CSC-31	m	40	-0.25	0	12		-0.25	0	12
CSC-32	m	47	0	0.1	10		0	0	10
CSC-33	f	46	0	-0.18	16		-0.5	-0.17609	15
CSC-34	m	53	-0.25	0	15		-0.25	0	15
CSC-35	m	36	-1	0.22	13		-1	0.04576	14
CSC-36	m	56	1.25	0.05	11		0.75	-0.07918	11
CSC-37	m	33	-1.5	-0.18	12		-2	-0.17609	12
CSC-38	m	39	-0.75	-0.18	12		-2.5	-0.17609	13
CSC-39	m	71	-0.5	0	12		-2	0	14
CSC-40	m	45	0.25	0	18		-2	-0.07918	18

CSC; Central serous choroiretinopathy,VA; visual acuity, IOP; intraocular pressure
