# Supplementary material for: Structural Changes of Inner and Outer Choroid in Central Serous Chorioretinopathy Determined by Optical Coherence Tomography
Source: PLoS One. 2016 Jun 15;11(6):e0157190. doi: 10.1371/journal.pone.0157190 (PMC4909210; doi:10.1371/journal.pone.0157190)
Supplement: S2 Table — (PDF) [file pone.0157190.s002.pdf]

Supplementary Data

S2 Table. Demographic findings of control eye.

Case number	sex	age	Refractive error	VA logMAR	IOP
C1	m	36	-1	-0.18	12
C2	m	37	-3.25	-0.18	16
C3	m	38	-4.5	0	19
C4	f	39	-5.5	-0.18	11
C5	f	39	-3.25	-0.18	12
C6	f	40	-5	-0.18	15
C7	m	40	-1.75	-0.18	14
C8	f	41	-0.75	-0.18	16
C9	m	41	-2.25	-0.18	10
C10	m	42	-2	-0.18	13
C11	f	43	-3.5	-0.08	14
C12	m	43	-2	-0.08	12
C13	f	43	-2.5	-0.3	14
C14	m	44	-3	-0.18	16
C15	m	45	0	-0.18	12
C16	f	47	-0.5	-0.18	16
C17	f	48	-1	-0.18	15
C18	m	50	-2.75	-0.08	13
C19	f	51	-1.75	-0.3	14
C20	m	54	-0.5	-0.3	11

C21	m	60	-4.25	-0.18	16
C22	m	64	-2.25	-0.18	13
C23	m	44	-2	0	14
C24	m	49	-1.5	-0.18	11
C25	m	51	2.5	-0.18	10
C26	m	52	-0.5	-0.18	12
C27	f	50	0.25	-0.18	10
C28	f	52	-1.25	-0.08	15
C29	m	44	-2	-0.08	13
C30	m	32	-0.75	-0.18	13
C31	m	41	-4.25	-0.18	12
C32	m	47	-4.75	0	15
C33	m	45	0.5	0	18
C34	m	53	-3.25	-0.18	12
C35	m	36	0.75	-0.08	10
C36	f	55	-4	0	13
C37	m	33	-5	-0.18	11
C38	f	40	-3.5	-0.08	11
C39	m	71	-0.25	0	12
C40	f	45	-3	-0.18	12

CSC; Central serous choroiretinopathy,VA; visual acuity, IOP; intraocular pressure
