# Supplementary material for: Structural Changes of Inner and Outer Choroid in Central Serous Chorioretinopathy Determined by Optical Coherence Tomography
Source: PLoS One. 2016 Jun 15;11(6):e0157190. doi: 10.1371/journal.pone.0157190 (PMC4909210; doi:10.1371/journal.pone.0157190)
Supplement: S3 Table — (PDF) [file pone.0157190.s003.pdf]

Supplementary Data

S3 Table. Choroidal area of CSC eye.

Case number	Whole choroid (μm^2)			Inner Choroid (μm^2)			Outer Choroid (μm^2)			CSC index
	choroid area	hypo-refractive area	hyperrefractive area	choroid area	hypo-refractive area	hyperrefractive area	choroid area	hypo-refractive area	hyperrefractive area	
CSC-1	771700.446	618049.9907	153650.4553	170040.7568	122570.9498	47469.807	601659.6892	495479.0409	106180.6483	1.8
CSC-2	906701.526	668741.7525	237959.7735	140119.104	92601.648	47517.456	766582.422	576140.1045	190442.3175	1.55
CSC-3	543480.5086	383474.2751	160006.2335	105273.288	72076.11814	33197.16986	438207.2206	311398.1569	126809.0637	1.13
CSC-4	614785.7233	480077.2153	134708.508	88112.2372	64388.82773	23723.40947	526673.4861	415688.3875	110985.0985	1.38
CSC-5	844749.6373	689772.6235	154977.0137	168609.6503	125256.5747	43353.07555	676139.987	564516.0488	111623.9382	1.75
CSC-6	646543.485	528844.2075	117699.2775	78019.2	49330.89	28688.31	568524.285	479513.3175	89010.9675	3.13
CSC-7	734963.7358	483960.754	251002.9818	112701.7727	77711.16774	34990.605	622261.9631	406249.5863	216012.3768	0.84
CSC-8	636637.6397	457949.5395	178688.1002	111078.9104	82994.01573	28084.89464	525558.7293	374955.5237	150603.2056	0.84
CSC-9	912216.9919	739262.106	172954.8859	164974.7137	114243.6191	50731.0946	747242.2783	625018.4869	122223.7913	2.27
CSC-10	848047.1867	600886.3076	247160.879	147797.7185	97844.40883	49953.30966	700249.4682	503041.8988	197207.5694	1.3
CSC-11	678506.8599	520900.7615	157606.0984	114035.5097	84995.82303	29039.6867	564471.3501	435904.9384	128566.4117	1.15
CSC-12	849795.3431	659732.5999	190062.7433	141019.2394	90858.62174	50160.61766	708776.1037	568873.9781	139902.1256	2.24
CSC-13	1006772.76	824455.125	182317.635	200119.248	128052.495	72066.753	806653.512	696402.63	110250.882	3.55
CSC-14	503662.2335	366703.8625	136958.3711	82500.35047	42934.08953	39566.26093	421161.8831	323769.7729	97392.11014	3.06
CSC-15	638435.9899	420528.3641	217907.6257	77337.75087	50099.29774	27238.45314	561098.239	370429.0664	190669.1726	1.56
CSC-16	645592.1617	463938.3863	181653.7753	126242.0313	92424.888	33817.14332	519350.1303	371513.4983	147836.632	0.91
CSC-17	712058.4248	507453.5953	204604.8296	99150.73503	62768.18787	36382.54717	612907.6898	444685.4074	168222.2824	1.53
CSC-18	727744.2895	511491.4373	216252.8522	119498.131	83422.3777	36075.75333	608246.1585	428069.0596	180177.0989	1.02
CSC-19	516788.3445	363690.0225	153098.322	122833.9162	84947.1964	37886.71983	393954.4283	278742.8261	115211.6022	1.07
CSC-20	945140.7266	701071.6301	244069.0965	100853.9029	65851.146	35002.75687	844286.8238	635220.4841	209066.3396	1.61
CSC-21	824892.1253	622766.6693	202125.456	87782.049	47702.47133	40079.57767	737110.0763	575064.198	162045.8783	2.98

CSC-22	597899.52	412901.0007	184998.5193		80552.94735	55375.77197	25177.17539		517346.5726	357525.2287	159821.3439	1.02
CSC-23	875560.41	726890.9737	148669.4363		99007.758	60549.82633	38457.93167		776552.652	666341.1473	110211.5047	3.84
CSC-24	859429.9792	571939.6568	287490.3224		77832.279	64264.79426	13567.48474		781597.7002	507674.8626	273922.8376	0.39
CSC-25	644882.094	482810.093	162072.001		87141.98977	53262.15197	33879.8378		557740.1042	429547.941	128192.1632	2.13
CSC-26	698883.6093	480414.7952	218468.8142		99814.73067	64189.1745	35625.55617		599068.8787	416225.6207	182843.258	1.26
CSC-27	486410.8185	350810.082	135600.7365		99322.27283	60072.34583	39249.927		387088.5457	290737.7362	96350.8095	1.97
CSC-28	567619.2467	428816.298	138802.9487		93012.50866	62210.79166	30801.717		474606.738	366605.5063	108001.2317	1.68
CSC-29	706448.6081	551465.7893	154982.8189		89216.03854	58849.929	30366.10954		617232.5696	492615.8603	124616.7093	2.04
CSC-30	474583.6727	329650.9347	144932.738		91171.47233	68712.624	22458.84833		383412.2003	260938.3107	122473.8896	0.69
CSC-31	413998.0487	296876.0593	117121.9893		70818.98767	47712.22367	23106.764		343179.061	249163.8357	94015.22533	1.28
CSC-32	1089315.429	810075.1727	279240.2561		97033.63206	71119.99627	25913.63579		992281.7967	738955.1764	253326.6203	1.06
CSC-33	577151.6179	402660.8065	174490.8114		78466.20413	46986.0184	31480.18573		498685.4138	355674.7881	143010.6257	1.67
CSC-34	795980.052	530017.398	265962.654		112476.906	74812.518	37664.388		683503.146	455204.88	228298.266	1
CSC-35	560719.985	411050.1346	149669.8504		74970.414	45411.29597	29559.11803		485749.571	365638.8386	120110.7324	1.98
CSC-36	488189.3543	403670.1023	84519.252		88805.664	57415.939	31389.725		399383.6903	346254.1633	53129.527	3.56
CSC-37	675924.4477	459786.8438	216137.604		81452.12247	49631.43413	31820.68834		594472.3253	410155.4096	184316.9156	1.42
CSC-38	720624.0824	544887.042	175737.0404		141769.938	96361.07607	45408.86197		578854.1443	448525.9659	130328.1784	1.62
CSC-39	829122.748	579228.5773	249894.1706		134368.0807	91221.96699	43146.11367		694754.6673	488006.6103	206748.057	1.11
CSC-40	512094.1121	413111.8383	98982.27386		109102.7109	77619.3514	31483.35947		402991.4013	335492.4869	67498.9144	2.02
average	702101.3494	520020.3706	182080.9788	#	109110.9218	73471.35109	35639.57073		592990.4275	446549.0195	146441.408	1.7
SD	160651.9064	132420.0951	49484.13248	#	30664.77349	22797.27463	10229.34473		146632.167	121214.2774	49822.1854	0.83

CSC. Central serous chorioretinopathy.
