# Supplementary material for: Structural Changes of Inner and Outer Choroid in Central Serous Chorioretinopathy Determined by Optical Coherence Tomography
Source: PLoS One. 2016 Jun 15;11(6):e0157190. doi: 10.1371/journal.pone.0157190 (PMC4909210; doi:10.1371/journal.pone.0157190)
Supplement: S4 Table — (PDF) [file pone.0157190.s004.pdf]

Supplementary Data

S4 Table. Choroidal area of CSC fellow eye.

Case number	Whole choroid (μm^2)				Inner Choroid (μm^2)				Outer Choroid (μm^2)			CSC index
	choroid area	hypo-refractive area	hyperrefractive area		choroid area	hypo-refractive area	hyperrefractive area		choroid area	hypo-refractive area	hyperrefractive area	
CSC-1	495787.8091	350142.449	145645.3601		124218.4087	104456.389	19762.01969		371569.4004	245686.0599	125883.3404	0.37
CSC-2	483101.775	348563.16	134538.615		151806.555	114756.1425	37050.4125		331295.22	233807.0175	97488.2025	0.77
CSC-3	444163.9246	330299.2366	113864.688		82912.73753	66589.89337	16322.84416		361251.1871	263709.3432	97541.84384	0.66
CSC-4	307988.2999	205558.7844	102429.5155		87396.86793	68817.19159	18579.67634		220591.4319	136741.5928	83849.83914	0.44
CSC-5	803531.583	638453.637	165077.946		157683.15	117033.5215	40649.62853		645848.433	521420.1155	124428.3175	1.46
CSC-6	656450.8785	504979.0785	151471.8		75627.42916	60244.0345	15383.39466		580823.4493	444735.044	136088.4053	0.83
CSC-7	752300.4842	578338.9726	173961.5116		89125.84847	71569.0904	17556.75807		663174.6357	506769.8822	156404.7535	0.79
CSC-8	686474.0096	519630.6857	166843.3239		106107.834	73556.21916	32551.61487		580366.1755	446074.4665	134291.709	1.47
CSC-9	511147.5683	348869.4512	162278.1171		128340.4616	101769.1188	26571.3428		382807.1067	247100.3324	135706.7743	0.48
CSC-10	534737.2114	412730.8171	122006.3943		102627.6396	79561.28974	23066.34988		432109.5718	333169.5274	98940.04446	0.98
CSC-11	651855.8144	459791.3524	192064.4619		102357.7075	77820.08156	24537.62598		549498.1068	381971.2709	167526.836	0.72
CSC-12	699532.5311	490522.6549	209009.8763		149167.602	106016.328	43151.274		550364.9291	384506.3269	165858.6023	0.94
CSC-13	917061.3609	770137.7982	146923.5627		126531.6429	99003.58835	27528.05454		790529.718	671134.2099	119395.5081	1.56
CSC-14	314766.837	200450.4232	114316.4138		76143.89483	52587.7855	23556.10933		238622.9422	147862.6377	90760.3045	0.73
CSC-15	609394.2132	393185.4789	216208.7343		87119.81486	59959.2064	27160.60845		522274.3983	333226.2725	189048.1258	0.79
CSC-16	526156.495	358424.6375	167731.8575		115158.1965	86075.835	29082.3615		410998.2985	272348.8025	138649.496	0.66
CSC-17	525230.5195	369716.604	155513.9155		89098.1665	69338.858	19759.3085		436132.353	300377.746	135754.607	0.63
CSC-18	706240.462	549508.408	156732.054		88519.2705	76420.432	12098.8385		617721.1915	473087.976	144633.2155	0.52
CSC-19	764919.304	585050.0985	179869.2055		90185.8475	69103.158	21082.6895		674733.4565	515946.9405	158786.516	0.99
CSC-20	486725.8305	315258.9525	171466.878		98716.541	75740.3845	22976.1565		388009.2895	239518.568	148490.7215	0.49
CSC-21	807600.483	666685.885	140914.598		95090.495	65425.418	29665.077		712509.988	601260.467	111249.521	2.45

CSC-22	693693.4755	529523.485	164169.9905		115003.1145	89036.0975	25967.017		578690.361	440487.3875	138202.9735	0.93
CSC-23	647045.284	495911.749	151133.535		83053.772	63688.099	19365.673		563991.512	432223.65	131767.862	1
CSC-24	553692.713	400954.383	152738.33		109619.358	79406.268	30213.09		444073.355	321548.115	122525.24	0.99
CSC-25	368931.1425	236633.0605	132298.082		71515.4735	56414.0665	15101.407		297415.669	180218.994	117196.675	0.41
CSC-26	562015.8575	382726.518	179289.3395		83720.5355	80205.267	3515.2685		478295.322	302521.251	175774.071	0.08
CSC-27	433577.075	289738.2295	143838.8455		55789.6205	48389.408	7400.2125		377787.4545	241348.8215	136438.633	0.27
CSC-28	396555.1955	273703.049	122852.1465		77126.1	53337.9365	23788.1635		319429.0955	220365.1125	99063.983	0.99
CSC-29	722249.8295	578591.9505	143657.879		93876.1295	68409.61	25466.5195		628373.7	510182.3405	118191.3595	1.61
CSC-30	865898.126	733914.818	131983.308		105827.612	78313.747	27513.865		760070.514	655601.071	104469.443	2.2
CSC-31	584498.5115	431617.791	152880.7205		87625.14	74521.0785	13104.0615		496873.3715	357096.7125	139776.659	0.45
CSC-32	657640.0095	446927.099	210712.9105		81697.306	67626.1045	14071.2015		575942.7035	379300.9945	196641.709	0.4
CSC-33	394706.221	253040.128	141666.093		72337.1555	50813.2375	21523.918		322369.0655	202226.8905	120142.175	0.71
CSC-34	343215.807	253697.8105	89517.9965		57408.037	47078.1755	10329.8615		285807.77	206619.635	79188.135	0.57
CSC-35	474091.7625	356343.6265	117748.136		62958.394	48657.718	14300.676		411133.3685	307685.9085	103447.46	0.87
CSC-36	419331.8444	296984.522	122347.3224		82419.03614	55286.84044	27132.1957		336912.8082	241697.6815	95215.12669	1.25
CSC-37	691882.9214	530175.1631	161707.7583		74620.51037	42548.53862	32071.97175		617262.411	487626.6245	129635.7865	2.84
CSC-38	564542.6274	396467.442	168075.1854		111378.8963	85546.86346	25832.03281		453163.7312	310920.5786	142243.1526	0.66
CSC-39	735395.8576	526232.5519	209163.3057		131758.4274	100027.7654	31730.66196		603637.4302	426204.7865	177432.6437	0.76
CSC-40	439172.5174	321561.7468	117610.7706		87530.65468	67710.77034	19819.88434		351641.8627	253850.9765	97790.88625	0.76
average	580832.6043	428276.0922	152556.5121	#	96730.0346	73821.53894	22908.49566	#	484102.5697	354454.5533	129648.0164	0.91
SD	155546.5508	141512.3523	29996.22461	#	24579.39683	19007.52537	8521.641405	#	146469.8035	135816.089	28847.02916	0.57

CSC. Central serous chorioretinopathy.
