# Supplementary material for: Structural Changes of Inner and Outer Choroid in Central Serous Chorioretinopathy Determined by Optical Coherence Tomography
Source: PLoS One. 2016 Jun 15;11(6):e0157190. doi: 10.1371/journal.pone.0157190 (PMC4909210; doi:10.1371/journal.pone.0157190)
Supplement: S5 Table — (PDF) [file pone.0157190.s005.pdf]

Supplementary Data

S5 Table. Choroidal area of control eye.

Case number	Whole choroid (μm^2)				Inner Choroid (μm^2)				Outer Choroid (μm^2)			CSC index
	choroid area	hypo-refractive area	hyperrefractive area		choroid area	hypo-refractive area	hyperrefractive area		choroid area	hypo-refractive area	hyperrefractive area	
C1	603550.8423	456474.627	147076.215		79396.72667	62816.1566	16580.57007		524154.1156	393658.4704	130495.6452	0.79
C2	318246.354	209629.773	108616.581		85189.923	64685.889	20504.034		233056.431	144943.884	88112.547	0.52
C3	536532.93	355725.6479	180807.282		119398.9815	89329.0815	30069.9		417133.9485	266396.5664	150737.3821	0.59
C4	580092.6053	353737.3914	226355.214		95656.08448	72761.37832	22894.70616		484436.5209	280976.013	203460.5078	0.43
C5	559999.5263	380510.784	179488.742		101068.3009	69102.72	31965.58087		458931.2254	311408.064	147523.1614	0.98
C6	512718.0723	350304.273	162413.799		88405.85427	70464.53427	17941.32		424312.218	279839.7387	144472.4793	0.49
C7	338732.4892	240921.1418	97811.3473		68629.806	54987.08834	13642.71766		270102.6832	185934.0535	84168.62968	0.55
C8	656236.8287	413407.9725	242828.856		136079.4167	107240.796	28838.62073		520157.412	306167.1765	213990.2355	0.38
C9	282475.6538	169203.2692	113272.385		93829.40751	64488.61583	29340.79168		188646.2463	104714.6533	83931.59298	0.57
C10	295561.188	203365.5973	92195.5907		87583.42117	65550.15683	22033.26434		207977.7668	137815.4405	70162.32631	0.66
C11	659848.545	482035.5514	177812.994		112676.6751	75743.02093	36933.6542		547171.8699	406292.5305	140879.3394	1.41
C12	475996.5326	322843.6043	153152.928		92722.104	79667.20086	13054.90314		383274.4286	243176.4034	140098.0252	0.28
C13	455089.7087	318083.6593	137006.049		87962.31347	66491.5538	21470.75967		367127.3953	251592.1055	115535.2898	0.7
C14	343658.9605	230265.3676	113393.593		98070.13428	71971.60888	26098.5254		245588.8263	158293.7587	87295.06753	0.66
C15	340236.7003	222095.662	118141.038		95438.03149	71359.08467	24078.94682		244798.6688	150736.5773	94062.0915	0.54
C16	517684.8845	340899.6187	176785.266		97418.48832	60685.85997	36732.62835		420266.3962	280213.7587	140052.6374	1.21
C17	349324.1322	220982.7337	128341.399		64094.37027	48894.03833	15200.33194		285229.7619	172088.6953	113141.0666	0.47
C18	590088.9683	389712.2569	200376.711		79991.65527	67382.91686	12608.73841		510097.313	322329.34	187767.9729	0.32
C19	290359.4037	174270.1869	116089.217		65448.92852	51379.40551	14069.52301		224910.4752	122890.7814	102019.6938	0.33
C20	281397.7187	194924.1723	86473.5464		67707.62543	57869.18128	9838.444153		213690.0933	137054.991	76635.10224	0.3
C21	237766.8557	150134.6871	87632.1686		68424.51024	59020.29885	9404.211394		169342.3454	91114.38823	78227.95721	0.19

C22	523392.4163	372318.2665	151074.15		84901.90515	73019.1726	11882.73255		438490.5111	299299.0939	139191.4172	0.35
C23	476684.1604	384391.7563	92292.4041		81148.40157	61346.53399	19801.86758		395535.7588	323045.2223	72490.53651	1.44
C24	595888.4143	470791.6487	125096.766		73999.65094	54899.38035	19100.27059		521888.7633	415892.2684	105996.495	1.37
C25	580149.8793	395618.8678	184531.012		84877.75661	62009.36275	22868.39385		495272.1227	333609.505	161662.6177	0.76
C26	393526.6157	268509.7642	125016.852		72308.38032	61227.23904	11081.14128		321218.2354	207282.5251	113935.7102	0.33
C27	579806.7437	417022.8406	162783.903		101128.5802	76565.99968	24562.58048		478678.1635	340456.841	138221.3226	0.79
C28	396597.2285	287900.879	108696.349		95628.412	78001.36704	17627.04496		300968.8165	209899.512	91069.30448	0.52
C29	189569.9376	116809.6032	72760.3344		85150.5264	65304.8568	19845.6696		104419.4112	51504.7464	52914.6648	0.3
C30	415162.6218	284895.8394	130266.782		84330.95328	56800.6896	27530.26368		330831.6685	228095.1498	102736.5187	1.08
C31	394083.3382	269928.6922	124154.646		76760.76896	59772.55232	16988.21664		317322.5693	210156.1398	107166.4294	0.56
C32	598915.7821	398238.4182	200677.364		76544.35872	60118.80928	16425.54944		522371.4234	338119.609	184251.8144	0.5
C33	567232.7976	370543.4971	196689.301		108510.6636	70020.33731	38490.32631		458722.134	300523.1598	158198.9742	1.04
C34	649256.4634	443300.4502	205956.013		76517.60881	56450.32784	20067.28097		572738.8546	386850.1224	185888.7321	0.74
C35	426687.4136	294159.9134	132527.5		96029.17066	78195.4864	17833.68425		330658.2429	215964.427	114693.8159	0.43
C36	360959.4138	211006.2144	149953.199		75783.76416	50402.92966	25380.8345		285175.6496	160603.2847	124572.3649	0.65
C37	255591.9396	167375.853	88216.0866		73513.40544	64102.80384	9410.6016		182078.5341	103273.0492	78805.48499	0.19
C38	434391.0525	304792.4925	129598.56		62240.2425	50112.63	12127.6125		372150.81	254679.8625	117470.9475	0.52
C39	489627.5816	331798.0295	157829.552		127403.709	94620.76461	32782.94435		362223.8727	237177.2649	125046.6078	0.66
C40	161949.6317	98896.01149	63053.6202		43869.48974	35765.41958	8104.070165		118080.1419	63130.59192	54949.55001	0.26
average	442876.8083	301695.6754	141181.133		86646.01267	66015.68123	20630.33143		356230.7956	235679.9941	120550.8015	0.62
SD	137152.8635	101640.4983	43338.5554		18200.36709	12998.31643	8128.139631		128927.8932	97351.76974	40045.35074	0.33
